# Crosstalk between microbial biofilms in the gastrointestinal tract and chronic mucosa diseases

**DOI:** 10.3389/fmicb.2023.1151552

**Published:** 2023-04-13

**Authors:** Yumeng Wang, Shixi Xu, Qiurong He, Kun Sun, Xiaowan Wang, Xiaorui Zhang, Yuqing Li, Jumei Zeng

**Affiliations:** ^1^West China-PUMC C.C. Chen Institute of Health, West China School of Public Health and West China Fourth Hospital, Sichuan University, Chengdu, China; ^2^State Key Laboratory of Oral Diseases, National Clinical Research Center for Oral Diseases, West China Hospital of Stomatology, Sichuan University, Chengdu, Sichuan, China

**Keywords:** biofilm, gastrointestinal tract, mucosa disease, microbiota, crosstalk

## Abstract

The gastrointestinal (GI) tract is the largest reservoir of microbiota in the human body; however, it is still challenging to estimate the distribution and life patterns of microbes. Biofilm, as the predominant form in the microbial ecosystem, serves ideally to connect intestinal flora, molecules, and host mucosa cells. It gives bacteria the capacity to inhabit ecological niches, communicate with host cells, and withstand environmental stresses. This study intends to evaluate the connection between GI tract biofilms and chronic mucosa diseases such as chronic gastritis, inflammatory bowel disease, and colorectal cancer. In each disease, we summarize the representative biofilm makers including *Helicobacter pylori*, adherent-invasive *Escherichia coli*, *Bacteroides fragilis*, and *Fusobacterium nucleatum*. We address biofilm’s role in causing inflammation and the pro-carcinogenic stage in addition to discussing the typical resistance, persistence, and recurrence mechanisms seen *in vitro*. Biofilms may serve as a new biomarker for endoscopic and pathologic detection of gastrointestinal disease and suppression, which may be a useful addition to the present therapy strategy.

## 1. Introduction

Biofilms are complex three-dimensional structures formed by an aggregate of microorganisms embedded in a self-produced matrix of extracellular polymeric substances (EPSs) that adhere to the biological or non-biological surface (Hall-Stoodley et al., 2004; López et al., 2010; Vert et al., 2012). By growing on the surfaces of medical equipment, implants, and exposed surfaces such as wounds and upper respiratory tracts, biofilms are infamous in the clinical setting for causing iatrogenic infection and chronic infection (Pintucci et al., 2010; Percival et al., 2015; Diaconu et al., 2018). In contrast to free-living cells, biofilm cells have a tendency to develop resistance to harsh environments and increase their tolerance to stressors, including starvation, dehydration, and antimicrobial agents (Rumbaugh and Sauer, 2020). Because of this, biofilms act as bacterial fortifications to withstand stress and maintain bacteria’s biological activity.

Bacterial colonization in the GI tract is more complicated due to immune system surveillance, interspecies competition, biophysical variables, and other reasons (Motta et al., 2019; Arias and Brito, 2021). Both commensals and pathogens are supposed to form biofilms when they colonize and occupy an ecological niche, but in healthy populations, the biofilm typically forms over the mucus layer, whereas in disease conditions, biofilms will penetrate the mucus and directly interact with the epithelial cells (Motta et al., 2021). One noteworthy occurrence is the advanced development of biofilm communities, even though the total number and diversity of bacteria frequently decline during gastrointestinal illness (Dejea et al., 2014). Since disease progression and biofilm density are positively correlated, biofilms are considered to constitute a “tipping point” between health and disease (Tytgat et al., 2019).

**Figure 1:**
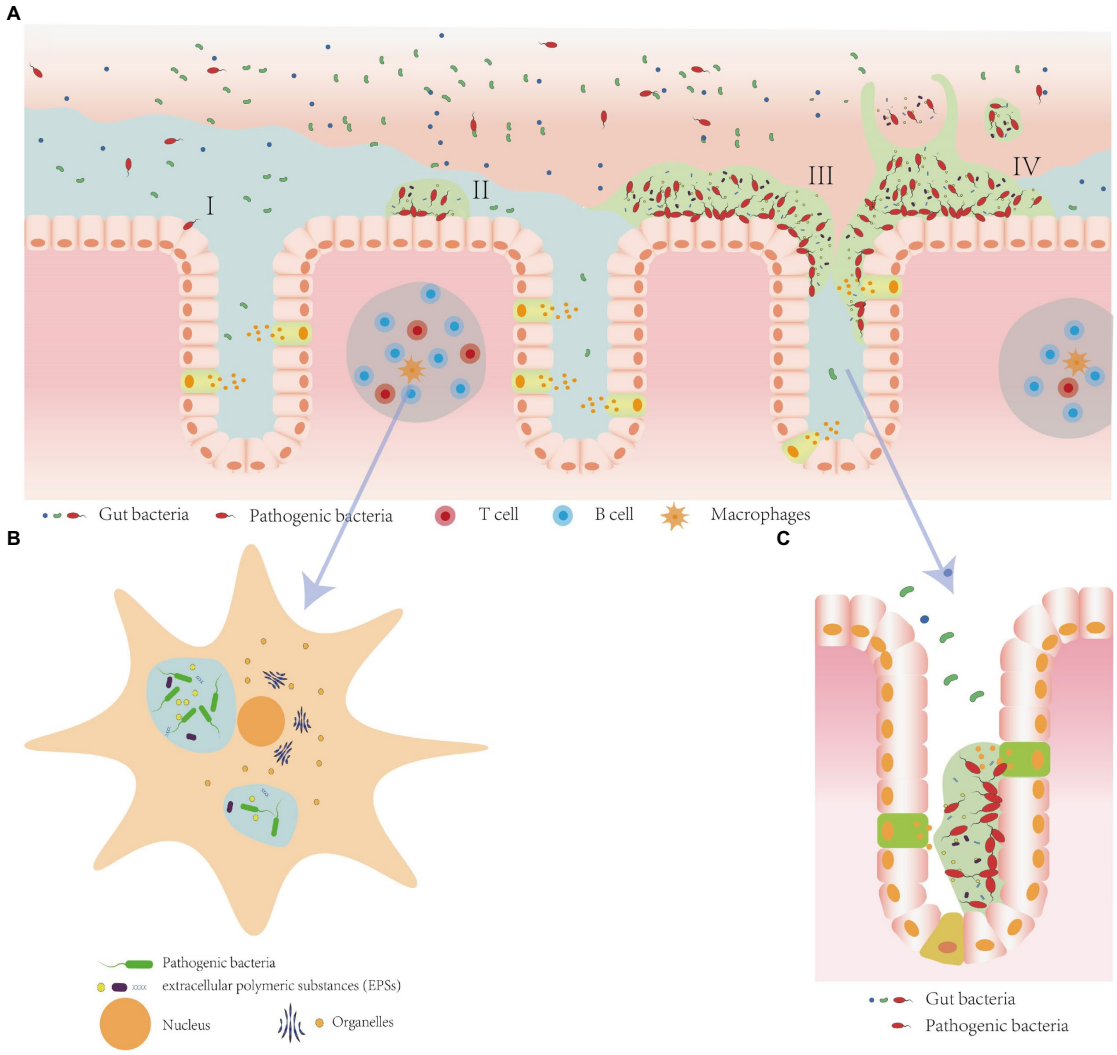
Biofilm formation on the mucosa layer, inside Macrophages, and within crypts. **(A)** Biofilm formation process. (I) Adhesion: Planktonic bacteria form reversible adhesion to the mucosa layer by van der Waals forces (Donlan, 2002; Garrett et al., 2008). (II) Colonization: The primary colonizing bacteria colonize the niche with the help of their extracellular secretions and unique features such as flagella and mycelium, continuously produce and secret exopolysaccharides and other substances to form extracellular polymeric substance (EPS) containing exopolysaccharides, extracellular proteins, extracellular DNA, and water (Dufour et al., 2010; Flemming and Wingender, 2010). (III) Maturation: Bacterial community multiplies and build a multidimensional structure. (IV) Dispersal: Bacteria spread and colonize other niches due to nutrient limitation or continued infestation (Hall-Stoodley et al., 2004). **(B)** Intracellular biofilm-like structure formed by bacteria such as *adherent-invasive E. coli* (Glasser et al., 2001). **(C)** Biofilms inside the intestinal crypt (Raskov et al., 2018).

According to earlier research, drug resistance and environmental tolerance are the main consequences of biofilm formation in single bacteria models *in vitro* (Sharma et al., 2019; Uruén et al., 2020). In the GI tract, biofilm formation might be a proactive tactic, rather than a reaction to stresses. For instance, to encourage co-colonization, biofilm communities form polybacterial structures and inter-species cross-feeding systems (Dejea et al., 2018). In addition, the identification of intestinal crypt biofilm and intracellular structures resembling biofilms suggested that biofilms interfere with host cell function and result in chronic inflammation (Swidsinski et al., 2005; Martinez-Medina et al., 2009; Prudent et al., 2021). It is useful to assess pathogenicity considering biofilm formation capacity since it represents both invasion effectiveness and persistence. To offer new insights into illness diagnosis and treatment, we describe representative biofilm producers in GI chronic mucosa disease and highlight their roles as initiators or mediators (Figure 1).

## 2. Microbial biofilms and gastrointestinal tract chronic disease

### 2.1. Chronic gastritis

Chronic gastritis is the primary cause of peptic ulcers and gastric cancers in most cases (Sipponen and Maaroos, 2015). Strong links exist between *Helicobacter pylori* and gastric inflammation in the development of gastric malignancy (Neng et al., 2013). Childhood *H. pylori* infection is a well-established etiological factor (in more than 90% of the cases) for the development of adult chronic gastritis (Pacifico et al., 2010; Kalach et al., 2017). However, research on the pathogenicity of *H. pylori* biofilm is still uncomplicated. In individuals with peptic ulcers and urease positivity, the entogastric *H. pylori* biofilm was initially photographed in the gastric mucosa in 2006 (Carron et al., 2006; Coticchia et al., 2006). *H. pylori* biofilm only forms at the air–liquid interface because of its microaerobic and capnophilic nature; therefore, the stomach is an ideal environment for the growth of biofilms (Cole et al., 2004; García et al., 2014). *H. pylori* cells in biofilm go through morphological changes and eventually enter coccoid status (the dormant state of *H. pylori*) under the prolonged culture *in vitro* (Krzyzek et al., 2019; Krzyzek and Grande, 2020), indicating that the biofilm renders *H. pylori* to tolerate and survive under challenging environmental circumstances. While the majority of *H. pylori* found in patients are responsive to treatments, a tiny subset of bacteria may resist their effects, causing infection to continue (Lewis, 2007). *H. pylori* recovers from the coccoid to the spiral form as the antibiotic concentration drops, repopulating the biofilm or causing diffusion (Zhang, 2014).

In an *in vitro H. pylori* biofilm model, outer membrane vesicles (OMVs) have been shown to participate in the colonization and stabilization of biofilms mediated by G^−^ bacilli during host infection (Fiocca et al., 1999). Further studies discussed the role of *H. pylori*-derived OMVs in biofilm formation, stabilization, and infection (Schooling and Beveridge, 2006), as well as the release of bacterial cytotoxins (Fiocca et al., 1999). A recent study reported a special strain TK1402, which exhibited a strong biofilm-forming ability with higher production of OMV (Jarzab et al., 2020), suggesting that the biofilm-formation ability of *H. pylori* may depend on OMVs-mediated bacterial-to-bacterial contact (Yonezawa et al., 2009). In chronic gastritis, the *H. pylori* biofilm serves as a physical barrier to external factors, and it modifies its surface structure in response to medicines such as calcitonin, which improves environmental adaptation (Gaddy et al., 2015). In addition, biofilm allows for limited reactive oxygen species (ROS) penetration, which may partially account for phagocytic cells’ inability to eradicate bacteria that are developing in biofilms (Costerton et al., 1999).

*Helicobacter pylori* is known to exhibit increased resistance to antibiotics when it is in a biofilm state. This might be due to the high expression of the lux gene mutation within biofilms, which regulates quorum sensing and produces extracellular molecular signals linked to the autoinducer AI-2 (cyclicfuranone; Lee et al., 2006). The chemical drive of AI-2 impacts the spatial organization and diffusion of *H. pylori* within the biofilm (Anderson et al., 2015). Simultaneously, the *lux* gene activates the methyl cycle, generates S-adenosylmethionine required by methyltransferases, and recycles cell products through methionine and intercellular signaling (Doherty et al., 2010). These processes, in turn, facilitate the regulation of signaling within the biofilm. Moreover, with higher expression of proton pump genes in biofilms than in the planktonic form, *H. pylori* shows enhanced resistance to clarithromycin, amoxicillin, and metronidazole. This shows that the efflux pump and biofilm increase drug resistance in concert (Attaran et al., 2017; Ge et al., 2018; Yonezawa et al., 2019). Furthermore, eDNA can promote microbial adhesion and chelate cations to inhibit antibiotic diffusion (Okshevsky and Meyer, 2015). In addition to the abovementioned mechanisms, *H. pylori*’s surface endotoxin and outer membrane structure can be altered by gene exchange during biofilm formation under the constant pressure of the immune system, ultimately leading to a decrease in *H. pylori* hydrophobicity (Gaddy et al., 2015), which further hinders drug penetration of biofilm.

Clinic information further supported the idea that biofilms and disease development were related. During the treatment of *H. pylori* patients, Cammarota et al. (2010) found that treatment of *H. pylori* patients with the biofilm-disrupting agent N-acetylcysteine (NAC) before conventional treatment resulted in the eradication of *H. pylori* in the experimental group, while the conventional treatment group did not exhibit the same outcome. In subsequent studies, it is crucial to specifically discuss strains when exploring the pathogenic mechanisms of *H. pylori* from a biofilm perspective because the formation of biofilm was independent of cell surface hydrophobicity and auto-aggregation but rather of the bacterial species itself, as well as the surrounding microenvironment of bacteria (Cole et al., 2004; Williams et al., 2008; Yonezawa et al., 2009, 2010; Hathroubi et al., 2020).

### 2.2. Inflammatory bowel disease – Ulcerative colitis and Crohn’s disease

In the realm of gastrointestinal diseases, Inflammatory bowel disease (IBD) is a commonly occurring condition comprising two distinct conditions: ulcerative colitis (UC) and Crohn’s disease (CD; McDowell et al., 2022). IBD patients typically have gut bacterial dysbiosis, which is defined by niche imbalance, a decline in commensal bacteria, and an increase in pathogenic germs. This condition can cause mucosal damage and immunological dysregulation (Kumar et al., 2019). Although the cause of the modification is yet unknown, external factors such as biofilms found during the endoscopic examination may be used as a guide and help with diagnosis and treatment.

A research of 1,426 individuals with UC and irritable bowel syndrome (IBS) found that these patients had a distinctive biofilm observation feature, with rates of 57% in IBS patients and 34% in UC patients compared with 6% of the controls (Baumgartner et al., 2021). In addition, patients who tested positive for biofilms had higher levels of calprotectin and a buildup of bile acids in the biofilms, which strongly suggested more severe inflammation and diarrhea. At the microscopic level, IBD patients’ biopsy specimens from the ileum, ascending colon, and sigmoid colon revealed a significantly greater incidence of bacteria concentration—93% against 35% in healthy controls (Swidsinski et al., 2008). With further information from clinical studies, biofilm may develop into a more representative marker in endoscopy and biopsy signaling susceptibility groups and disease severity.

One intriguing point of IBD is the transition of microbial communities, which comprises a considerable decrease in the total quantity of microbiota and a shift in the dominant flora from obligately anaerobic bacteria to facultatively anaerobic bacteria (Table 1). Despite the lack of clarity regarding the cause-and-effect relationship between dysbiosis and IBD, this alternation promotes GI tract instability and inflammation.

**Table 1:** Alternations of microbiota portion in IBD (UC and CD) patients.

		UC		CD		Reference
*Firmicutes*	↓	*Lachnospiraceae* (*Clostridia clusters XIVa*; *Clostridia clusters XIVb*)	↓	*Lachnospiraceae*	↓↓	1,5
				*Roseburia hominis*	↓	2
				*Faecalibacterium prausnitzii*	↓↓	2,3
		*Ruminococcus* (*Ruminococcus torques*; *Ruminococcus gnavus*)	↑	*Ruminococcus* (*Ruminococcus torques*; *Ruminococcus gnavus*)	↑	2,3
		*Clostridium* (*Clostridium hathewayi*; *Clostridium bolteae*; *Clostridioides difficile*)	↑	*Clostridium* (*Clostridium hathewayi*; *Clostridium bolteae*)	↑	2–4,6
				Coprococcus	↓	2
Bacteroidetes	↓↓	*Prevotella*	↑↓	*Prevotella*	↑↓	4
Proteobacteria	↑	*Enterobacteriaceae* (*Escherichia coli*)	↑	*Enterobacteriaceae* (*Escherichia coli*)	↑	1–4
Actinobacteria	↑					1
others		*Desulfovibrio*	↑			3,4
		*Akkermansia*	↓↓	*Akkermansia*	↓↓	4
				*Mycobacterium avium paratuberculosis*	↑	3,4,7
		*Campylobacter*	↑	*Campylobacter*	↑	4

↑: Bacterial taxa enriched in relative abundance; ↓: Bacterial taxa depleted in relative abundance; ↑↓: Inconsistent change in current reports. 1–7 = Frank et al. (2007), IBDMDB Investigators et al. (2019), Manichanh et al. (2012), Berry and Reinisch (2013), Gophna et al. (2006), Kang et al. (2010), and Feller et al. (2007).

In addition, we propose that the abrupt decline in probiotics, particularly those that produce butyrate, modifies the pattern of bacterial growth from dispersed to aggregated, which encourages the creation of biofilms. *In vitro* experiments showed that short-chain fatty acids (SCFAs) inhibit the formation of biofilms in species including *E.coli* (Amrutha et al., 2017), *Salmonella typhimurium* (Lamas et al., 2019), *Staphylococcus epidermidis* (Nakamura et al., 2020), and *Streptococcus gordonii* (Park et al., 2021). However, as IBD progress, active producers of SCFAs in health such as *Firmicutes* suffer great reduction. Moreover, it has been demonstrated that antibiotic treatment lowered SCFAs in the GI tract, which speeded up biofilm growth, morphogenesis, and GI colonization of *Candida albicans* (Guinan et al., 2019). Therefore, SCFAs are plausible natural inhibitors of biofilm formation which contribute to explaining the dual influence of antibiotics treatment on IBD patients.

Inflammatory bowel disease is currently believed to be the result of several variables working together in a synergistic way. Further research is required to determine whether microbiota play a role in the onset, but it is noteworthy to mention that a number of bacterial suspects in patients have a considerably increased capacity to produce biofilm. One possible pathogen that is frequently found in biopsies taken from IBD patients and involved in a biofilm community atop epithelial cells is *adherent-invasive E. coli* (*AIEC*; Darfeuille-Michaud et al., 2004). *AIEC* can penetrate the mucus layer by promoting mucin degradation with proteases (Kleessen et al., 2002). Then, a large portion of them outcompetes commensals in the gut by increasing the use of oxidized metabolites, which may trigger chronic inflammation and fibrosis. A minor portion, however, has been shown to invade and replicate in human macrophages and neutrophils in order to invade immune response or induce autophagy (Glasser et al., 2001; Small et al., 2013; Bretin et al., 2018; Xu et al., 2021). The capacity of biofilms to develop could link these processes. First, using *in vivo* RNA-sequencing, recent research revealed type IV secretion system (T4SS) in *AICE* significantly upregulated in the CD’s environment, which contribute to building biofilms on the epithelial cells’ surface (Elhenawy et al., 2021). In addition to adhesion and motility, biofilm may be associated with the expression of virulence genes such as sfa/focDE and ibeA (Martinez-Medina et al., 2009). Finally, the study on a CD-related strain *AIEC* LF82 showed macrophages are home to biofilm-like bacterial communities with a pathogenicity island HPI to secure the supply of iron, which constantly supply the extracellular portion following immune cell apoptosis (Prudent et al., 2021). Thus, the formation of biofilm allows AIEC to persist in the inflammation environment, accelerate mucosa damage, and create an immune cell-related chronic infection reservoir.

*Ruminococcus gnavus* is another essential component of biofilms in IBD patients enriched in both fecal and biopsy samples, and even abnormally proliferate in their unaffected relatives (Willing et al., 2010; Joossens et al., 2011). *R. gnavus* from the IBD samples express strain-specific genes for oxidative stress responses, adhesion, iron acquisition, and mucus utilization, thus guaranteeing an adaptive advantage in microbiota dysbiosis (Hall et al., 2017). It presents an intramolecular trans-sialidase to scavenge sialic acid from host mucus in a form, 2,7-anhydro-Neu5Ac, that can be utilized by themselves. Along with this process, the cross-feeding mechanism is strengthened as a result of the mucin core glycans’ exposure to other mucus-related bacteria (Crost et al., 2016; Bell et al., 2019). Therefore, *R. gnavus* is a key element in biofilm communities to alter the circumstances of the mucus layer and supply substrate to mucosa-associated bacteria (Png et al., 2010). *R. gnavus* found in CD produces an inflammatory polysaccharide that serves as a means of host–microbe interaction and encourages the production of inflammatory cytokines such as TNFα (Henke et al., 2019). However, because *R. gnavus* strains with a capsular polysaccharide elicit little to no TNFα secretion and hence promote a tolerogenic immune response, biofilm formation may weaken the pro-inflammation process (Henke et al., 2021). More focused isolation should be performed to determine whether encapsulated bacteria predominate in *R. gnavus*’s biofilm because capsular polysaccharide is regarded to be one of the key components in biofilms.

Although the causal relationship between other potential pathogens and IBD remains obscure, the ability of biofilm production might serve as an index to indicate the most related strains. A plausible pathogen of CD, for example, *Mycobacterium avium* spp. *paratuberculosis* (MAP) may be worth more attention since it can form thick biofilms in the environment though evidence of this is lacking *in vivo* (Cook et al., 2010; Patterson et al., 2021).

## 3. Colorectal cancer

For the total population, colorectal cancer (CRC) is the second-most lethal malignancy and the third-most frequent diagnosis (Dekker et al., 2019). Since gut microbial dysbiosis has been listed as an independent initiator for CRC, focus on the relationship between particular bacterial taxa and CRC progression has narrowed down the scope of potentially carcinogenic pathogens but failed to identify the most pertinent biological element (DeDecker et al., 2021). Biofilm, which combines microbiota composition and structure, is now recognized as a potential pro-carcinogenic stage (Dejea and Sears, 2016).

Although it seldom manifested in health, invasive polymicrobial bacterial biofilms were a distinctive hallmark of proximal CRC, present in approximately 89% of right-sided tumors. Since biofilm develops not only over the tumor mucosa but also over the nearby tumor-free mucosa, it may help sporadic CRC (Dejea et al., 2014). In addition, cell biological analysis shows its influence in increasing mucosal permeability and inflammation standards by decreasing epithelial cell E-cadherin and eliciting IL-6 and Stat3 activation (Dejea et al., 2014). Further evidence from murine models shows direct inoculation of biofilm-positive colon samples obtained from either healthy or sporadic CRC patients promoted tumorigenesis in three different genetic murine models of CRC by inducing early IL-17 and myeloid cell infiltration (Tomkovich et al., n.d.). This finding highlights that the biofilm community might trigger inflammation associated with colon tumor in the high-risk group.

The analysis based on biofilm communities also supplements the classical bacterial “driver–passenger” model, which classified microbiota involved in CRC into two main categories. The driver is defined as initial settlers that aggregate on and invade the mucus layer associated with the early stage of CRC but can be replaced later. The passenger is a relatively poor colonizer but has an advantage in the tumor microenvironment, which symbolizes the late stage of CRC (Tjalsma et al., 2012). We hypothesize that the development of biofilms could hasten the invasion of the drivers, serve as a platform for the exchange of chemicals with the host, and ensnare additional opportunistic pathogenic bacteria. Moreover, multi-bacterial symbiosis is more likely to take place with shared external substances, which creates a niche for opportunistic pathogens from the oral cavity.

Ideal drivers include bacterial taxa from *Bacteroides*, Actinomycetales, Enterobacteriaceae, and Ruminococcaceae (Tjalsma et al., 2012; Avril and DePaolo, 2021). *Bacteroides* are common commensals isolated from the biofilm community in IBD patients (Dejea et al., 2014), among which, *Bacteroides fragilis* is the most famous genus linked with tumorigenesis. For instance, enterotoxigenic *Bacteroides fragilis* (*ETBF*) is thought to reduce the mucus layer, impair the barrier function of the epithelial lining, and cause genotoxicity in host cells (Obiso et al., 1997; Li et al., 2017). The majority of these functions are attributable to the expression of *B. fragilis* toxin (BFT), a zinc-dependent metalloprotease that attracts type 17 T helper (Th17) cells and sets off an inflammatory cascade that is pro-cancerous and involves the signaling pathways IL-17R, NF-B, and Stat 3 (Chung et al., 2018). It is important to note that planktonic *ETBF* has higher BFT gene expression levels than biofilm formation strains, suggesting that biofilm may not always increase pathogenicity (Jasemi et al., 2020). However, mature biofilm may provide sustainable supplement for planktonic bacteria and exhibit stronger tolerance during chemotherapy and antibiotic treatment, which is prerequisite for long-term colonization. In addition, *ETBF* biofilms can assist the colonization of other drivers such as *pks* + *E.coli* by degrading mucus and dragging it from the colon lumen to the mucosa. *pks* + *E.coli* carries a genomic island coding for producing a putative hybrid peptide-polyketide genotoxin, which induces DNA double-strand break in human cells (Cuevas-Ramos et al., 2010). It is believed that co-colonization of *ETBF* and *pks* + *E.coli* in patients with familial adenomatous polyposis may enhance epithelial cells’ exposure to *pks* + *coil* and *BFT* and hasten the establishment of tumors due to the aggravation of interleukin-17 and DNA damage (Dejea et al., 2018).

According to studies, passengers contain both pro-inflammatory and anti-inflammatory taxa. Curiously, the pro-inflammatory taxonomic cluster is favorably connected with driver bacteria, whereas the anti-inflammatory taxa cluster is negatively correlated with driver bacteria (Geng et al., 2014). Although little is known about the process underlying this section, we believe that successful passengers might equip a better capacity for inducing inflammation and colonization to outcompete drivers. Growing members in oral bacteria are listed as passengers, such as *Fusobacterium*, *Peptostreptococcus*, *Porphyromonas*, and *Parvimonas* (Drewes et al., 2017; Wirbel et al., 2019; Liu et al., 2022). Oral bacteria can build co-abundance networks in colorectal tissues to form a strong link with the mucus layer and exhibit an imbalance in the gut microbiota of CRC patients (Flemer et al., 2018). The most representative biofilm maker is *Fusobacterium nucleatum*, which occupies a neutral position in the “driver–passenger” model and acts as a bridge between the initial drivers and the final passengers. *F. nucleatum* plays an essential role in structuring the polymicrobial biofilm model by bringing bacteria closer physically and increasing interspecies cross-talk, typically with *Streptococcus* species and *P. gingivalis* (Brennan and Garrett, 2019). Most virulence factors of *F. nucleatum* are adhesions, vital elements of biofilm that aid in the invasion of epithelial cells and co-colonization of other bacteria. For example, RadD adhesin and coaggregation CmpA-mediating protein are responsible for the binding of *S. gordonii* and *S. sanguinis* in the complex polymicrobial biofilm (Kaplan et al., 2009; Lima et al., 2017). FadA adhesin was found to bind to E-cadherin and further modulate E-cadherin/β-catenin signaling to induce adenomatous polyposis coli mutation (Rubinstein et al., 2013). Moreover, in both periodontal disease and CRC, FadA forms functional amyloid-like assemblies that serve as a scaffold to facilitate biofilm formation and confer acid tolerance (Meng et al., 2021; Tran et al., 2022). Studies of *F. nucleatum* as a sole initiator have shown that it promotes oncogenesis *via* a variety of pathways, including upregulating CRC cell glycolysis and activating TLR4 signaling to manipulate the expression of miRNA21, which results in the proliferation of CRC cells in the murine model (Yang et al., 2017, p. 21; Hong et al., 2021). On the contrary, research suggested that *F. nucleatum* alone may have an antitumor role in the murine model (Queen et al., 2022), which implied that its carcinogenic ability requires a specific consortium of cross-communicating or synergistic bacteria. We defined *F. nucleatum* as the fulcrum bacteria in CRC microecological dysfunction due to its ability to intensify the inflammatory milieu and draw pathogenic bacteria to a site. Understanding its biofilm structure can help us to construct a multifactorial modality cooperating in inducing oncogenesis and recurrence (Yu et al., 2017).

## 4. Discussion

The study of the interactions between the microbiota and the host from the perspective of biofilm is novel. Compared with fecal samples, it depicts a more realistic microbiota–epithelial cell interaction and highlights potential harmful microorganisms that were overlooked previously. For chronic gastritis, the etiological pointer for *H. pylori* is well established, and the underlying biofilm formation further explains the rapidly increasing rate of drug resistance and dissemination of *H. pylori*. For other multifactorial diseases including inflammatory bowel disease and colorectal cancer, biofilm may be an independent carcinogenic factor to induce inflammation and dysbiosis although the relevant molecular mechanisms have not been elucidated. We wish to underline that, in terms of the development of disease, the composition and structure of the microbiota may be equally essential. However, more biological samples are required to pinpoint the precise moment when bacteria begin to change into live organisms. Moreover, *in vivo* biofilm communities may be more active when different bacterial strains act as colonization starters, nutrition providers, or gene regulators for the epithelial cells. Using a polybacterial model in future studies may contribute to explaining bacterial interaction mechanisms in pathogenicity. Finally, biofilm is a viable therapeutic target that might be added to present antibiotic therapy to address the problem of bacterial drug resistance, which is on the rise. Dysbiosis may be improved by implanting probiotics that can successfully produce biofilms and outcompete the pathogen.

## Author contributions

YW and SX wrote the original draft with the help of QH. JZ reviewed and edited the manuscript with the help of YL. KS contributed to the schematic diagram. JZ and YL provided the conceptualization and funding. All authors contributed to the article and approved the submitted version.

## Funding

This study was supported by the International Research and Development Program of Sichuan (2022YFH0048 and 2021YFH0060) and the Fundamental Research Funds for the Central Universities (YJ201985).

## Conflict of interest

The authors declare that the research was conducted in the absence of any commercial or financial relationships that could be construed as a potential conflict of interest.

## Publisher’s note

All claims expressed in this article are solely those of the authors and do not necessarily represent those of their affiliated organizations, or those of the publisher, the editors and the reviewers. Any product that may be evaluated in this article, or claim that may be made by its manufacturer, is not guaranteed or endorsed by the publisher.
